# Systematic review and meta-analysis for the value of cardiac magnetic resonance strain to predict cardiac outcomes

**DOI:** 10.1038/s41598-023-50835-5

**Published:** 2024-01-11

**Authors:** Grigorios Korosoglou, Marios Sagris, Florian André, Henning Steen, Moritz Montenbruck, Norbert Frey, Sebastian Kelle

**Affiliations:** 1 Departments of Cardiology, Vascular Medicine and Pneumology, GRN Academic Teaching Hospital Weinheim, Roentgenstrasse 1, 69469 Weinheim, Germany; 2 Cardiac Imaging Center Weinheim, Hector Foundations, Weinheim, Germany; 3 Hippokration General Hospital, National and Kapodistrian University of Athens, School of Medicine, Athens, Greece; 4 Departments of Cardiology, Angiology and Pneumology, Heidelberg University, Heidelberg, Germany; 5 DZHK (German Centre for Cardiovascular Research), Partner Site Heidelberg/Mannheim, Heidelberg, Germany; 6 Department of Cardiology, Marien Hospital Hamburg, Hamburg, Germany; 7 Department of Cardiology, Angiology and Intensive Care Medicine, Deutsches Herzzentrum der Charité, Berlin, Germany; 8 DZHK (German Centre for Cardiovascular Research), Partner Site Berlin, Berlin, Germany

**Keywords:** Cardiology, Outcomes research

## Abstract

Cardiac magnetic resonance (CMR) is the gold standard for the diagnostic classification and risk stratification in most patients with cardiac disorders. The aim of the present study was to investigate the ability of Strain-encoded MR (SENC) for the prediction of major adverse cardiovascular events (MACE). A systematic review and meta-analysis was performed according to the PRISMA Guidelines, including patients with or without cardiovascular disease and asymptomatic individuals. Myocardial strain by HARP were used as pulse sequences in 1.5 T scanners. Published literature in MEDLINE (PubMed) and Cochrane’s databases were explored before February 2023 for studies assessing the clinical utility of myocardial strain by Harmonic Phase Magnetic Resonance Imaging (HARP), Strain-encoded MR (SENC) or fast-SENC. In total, 8 clinical trials (4 studies conducted in asymptomatic individuals and 4 in patients with suspected or known cardiac disease) were included in this systematic review, while 3 studies were used for our meta-analysis, based on individual patient level data. Kaplan–Meier analysis and Cox proportional hazard models were used, testing the ability of myocardial strain by HARP and SENC/fast-SENC for the prediction of MACE. Strain enabled risk stratification in asymptomatic individuals, predicting MACE and the development of incident heart failure. Of 1332 patients who underwent clinically indicated CMR, including SENC or fast-SENC acquisitions, 19 patients died, 28 experienced non-fatal infarctions, 52 underwent coronary revascularization and 86 were hospitalized due to heart failure during median 22.4 (17.2–28.5) months of follow-up. SENC/fast-SENC, predicted both all-cause mortality and MACE with high accuracy (HR = 3.0, 95% CI = 1.2–7.6, *p* = 0.02 and HR = 4.1, 95% CI = 3.0–5.5, respectively, *p* < 0.001). Using hierarchical Cox-proportional hazard regression models, SENC/fast-SENC exhibited incremental value to clinical data and conventional CMR parameters. Reduced myocardial strain predicts of all-cause mortality and cardiac outcomes in symptomatic patients with a wide range of ischemic or non-ischemic cardiac diseases, whereas in asymptomatic individuals, reduced strain was a precursor of incident heart failure.

## Introduction

Cardiovascular magnetic resonance (CMR) is the established reference standard for the identification of functional and structural abnormalities of the heart and for myocardial tissue characterization^1–4^. Hereby, a plethora of clinical questions such as the detection of myocardial ischemia due to coronary artery disease (CAD), differentiation of hypertrophy of unclear etiology, detection of subtle myocardial damage due to cardiotoxicity or infiltrative disorders can be addressed^5–9^.

**Figure 1:**
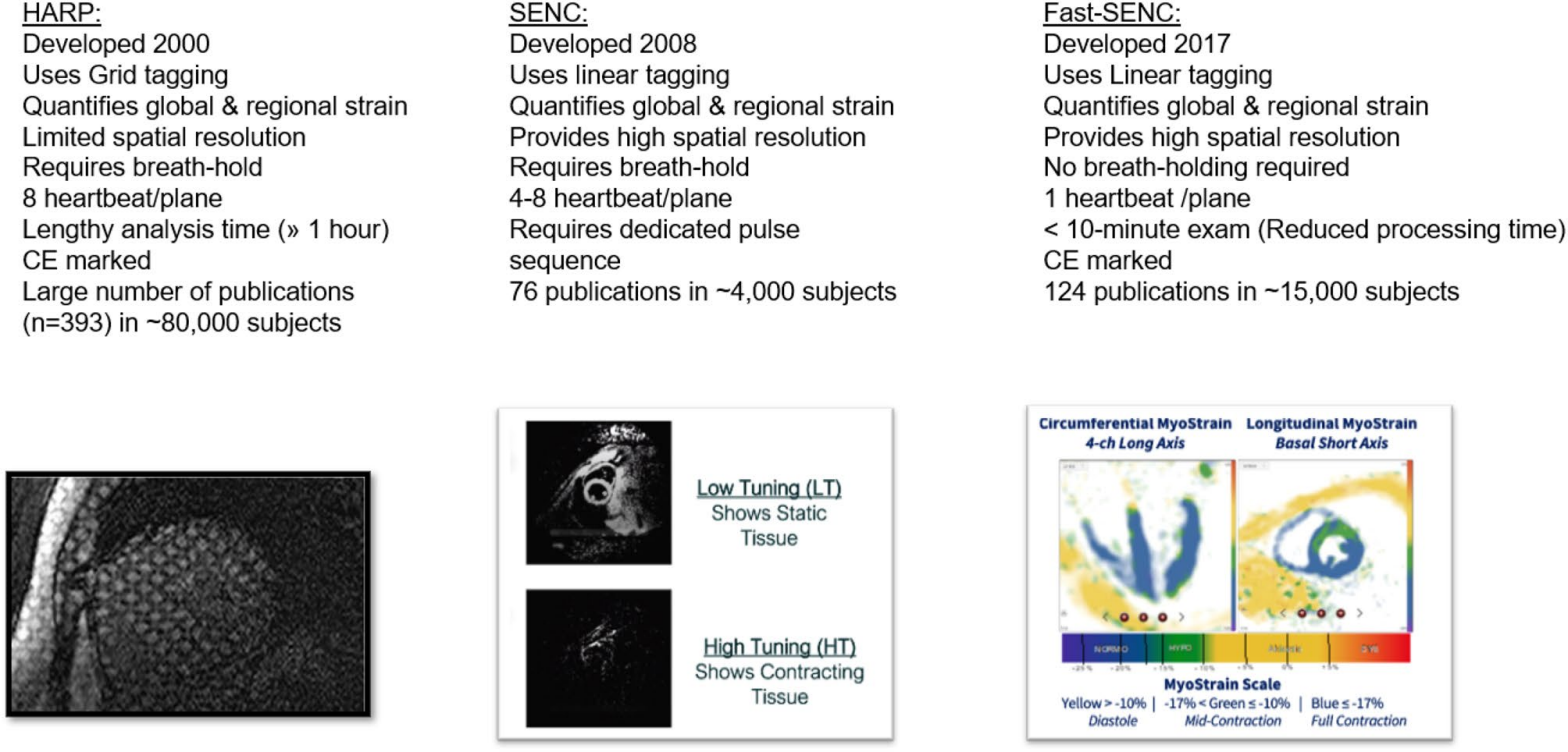
Overview of the technical features of HARP, SENC and fast-SENC sequences for the estimation of myocardial strain.

Harmonic phase strain analysis (HARP) for measuring myocardial strain was introduced in 2000^10,11^. However, HARP is time-consuming and has limited spatial resolution. Strain-encoded MR (SENC) arose within the last two decades, providing higher spatial resolution compared to HARP^12^. More recently, fast-SENC has been introduced, offering strain analysis within a single heartbeat, without the need for breath holding^13^. This has advantages in patients with arrhythmias and in those who cannot perform breathholds (Fig. 1). Experimental and clinical studies highlighted the role of SENC/fast-SENC in patients with different cardiac disorders^14^.

Recently, myocardial strain gained scientific and clinical interest for risk stratification of patients, i.e., the prediction of major adverse cardiovascular events (MACE)^14^, which constitutes a common endpoint in large randomized controlled trials (RCTs)^15^. While such ‘megatrials’ require the inclusion of large patient sample sizes to achieve a significantly altered primary endpoint, myocardial strain may serve as a surrogate marker, reducing the number of patients needing to be included^16^. We sought to systematically review clinical trials, investigating the prognostic role of strain by HARP, SENC and fast-SENC.

## Methods

### Eligibility criteria and study selection

We conducted our research in line with the recommendations of the Cochrane Collaboration Handbook and according to the PRISMA 2020 (Preferred Reporting Items for Systematic Reviews and Meta-Analyses, Supplementary Information) guidelines^17^. “CMR” and “Strain” combined with the AND/OR as Boolean comprised our search terms. PubMed, Google scholar and Cochrane’s search engine were utilized for our search. Our research strategy’s inclusion criteria were the following: (i) publications written and published in English language, (ii) patient populations consisting of adult patients with (symptomatic) or without (asymptomatic) cardiovascular disease undergoing CMR, including myocardial strain assessment using HARP, SENC or fast-SENC, (iii) studies reporting on MACE, including cardiovascular death, myocardial infarction, coronary revascularization and hospitalizations due heart failure and their association with CMR-based myocardial strain.

### Data extraction and statistical analysis

Based on individual patient data, which were available from 3 studies, investigating the role of SENC or fast-SENC on clinical outcomes^18–20^, we sought to determine the ability of myocardial strain for the prediction of all-cause mortality and MACE. All 3 studies included patients who underwent CMR due to clinical reasons, whereas studies including asymptomatic patients were not included. Statistics were performed using the dedicated statistical software (MedCalc 20.009, Mariakerke, Belgium). Continuous normally distributed variables were expressed as mean ± standard deviation, whereas non-normally distributed variables were reported as medians with interquartile range (IQR). Normal distribution was assessed using the Shapiro–Wilk test. Categorical variables were reported as numbers and proportions. For survival analysis the endpoints (i) all-cause mortality, and (ii) a composite endpoint, including all-cause mortality, myocardial infarction, coronary revascularization, and hospitalization due to heart failure were used. Survival curves were estimated by the Kaplan–Meier method to estimate the distribution of cardiac events as a function of the follow-up duration and comparisons were performed using log-rank tests. Cox proportional hazard models were used to evaluate the incremental value of myocardial strain to clinical and conventional CMR parameters. Furthermore, Cox proportional hazard models using the following hierarchic steps: (1) clinical data, (2) conventional CMR markers (wall motion abnormalities (WMA), late gadolinium enhancement (LGE) and LV-ejection fraction) and (3) myocardial strain by SENC or fast-SENC were applied. Model *χ*^2^ values were compared to each other for each incremental step. Differences were considered statistically significant at *p* < 0.05.

### Quality and risk of bias assessment

Bias assessment was performed according to the PROBAST tool, which is specialized for assessing the bias and applicability of studies developing prediction models^21^. Due to the character of the studies and the lack of validation in the general population, all studies include significant amounts of biases in the outcome domain. Moreover, the shrinking population especially for studies developing prediction models, may introduce additional biases in the analysis domain of the questionnaire. Therefore, the amount of bias insertion was evaluated as relatively high according to PROBAST^21^. On the other hand, the applicability of all studies was assessed with a reduced amount of bias for all studies since the software and hardware used for the studies is commercially available and such measures can be conducted in departments with available CMR equipment. The results of the risk of bias assessment are provided in Table 1.

**Table 1:** Bias assessment with PROBAST tool.

Studies	Participants	Predictors	Outcome	Analysis	Overall ROB	Overall applicability
Korosoglou et al. 2011^16^	Low	Low	High	High	High	Low
Choi et al. 2013^17^	Low	Low	High	High	High	Low
Sharma et al. 2014^18^	Low	Low	High	High	High	Low
Venkatesh et al. 2014^19^	Low	Low	High	High	High	Low
Mordi et al. 2015^20^	Low	Low	High	High	High	Low
Korosoglou et al. 2021^21^	Low	Low	High	High	High	Low
Steen et al. 2021^22^	Low	Low	High	High	High	Low
Pezel et al. 2022^23^	Low	Low	High	High	High	Low

## Results

**Figure 2:**
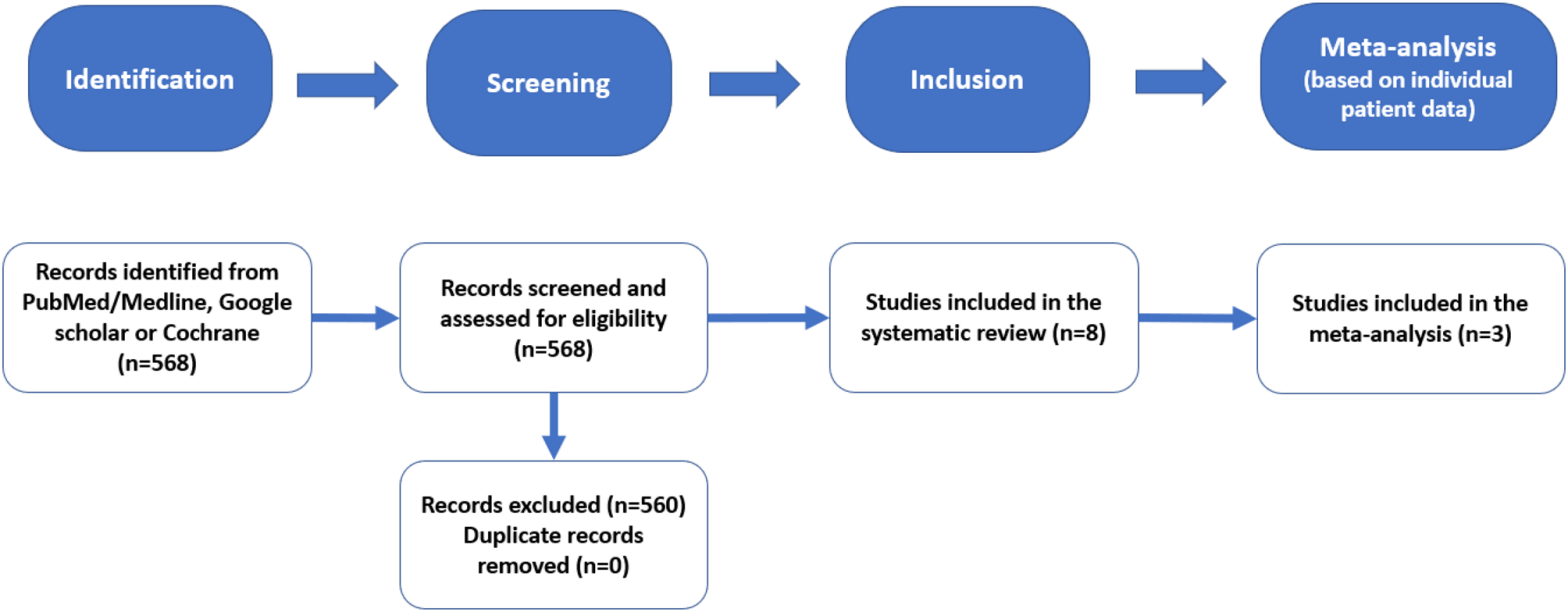
Prisma flow-chart for the studies included in our systematic review and meta-analysis.

Initially, 568 studies were identified based on predefined criteria. After exerting all inclusion and exclusion criteria 8 studies remained^18–20,22–26^. The selection strategy is depicted in the PRISMA Flowchart in Fig. 2. In total, 8349 patients were enrolled in these 8 studies, which were included in the final analysis. The follow-up duration ranged from 1.9–8.3 years (median = 2.3, IQR = 2.0–7.4 years). The basic characteristics of each study including population characteristics, endpoints, and CMR software and hardware are presented in Table 1. In addition, an overview of univariate and multivariable predictors of MACE and of the corresponding cut-off values for strain used in each study is provided in Tables 2, 3.

**Table 2:** Studies assessing strain based CMR by HARP, SENC or fast-SENC for the prediction of MACE.

Study	Population and follow-up duration	CMR prognostic parameters	MACE-Definition and cases recorded	Hardware and software
Korosoglou et al. 2011^18^	320 consecutive symptomatic patients with known or suspected CAD – Age: 64 ± 14, 74% males – Follow-up 2.3 ± 0.8 months	– WMA – Visual SENC – Strain reserve – Strain rate reserve	– Hard endpoints: 10 cardiac death, 25 nonfatal myocardial infarctions – 32 Revascularization > 90 days after CMR (27 PCI and 5 CABG)	– 1.5-T whole-body MR scanner Achieva system (Philips Medical Systems, Best, the Netherlands) – View Forum software (Philips Medical Systems, Best, the Netherlands) – Diagnosoft SENC, version 1.06 (Diagnosoft, Inc., Palo Alto, California)
Choi et al. 2013^22^	– MESA participants (asymptomatic individuals) from multiple ethnicities – 1768 patients with no CVD – Age: 64.9 ± 9.8, 53% males – Follow-up 5.5 ± 1.3 years	– HARP method – Ecc global and midmyocardial	– MESA endpoints: 39 newly HF diagnoses, 11 of which had MI. In total, 79 patients with CAD and 108 with newly diagnosed CVD	– 1.5 T scanners – HARP method analysis in MATLAB software or HARP1.15, Diagnosoft, Palo Alto, CA, USA
Sharma et al. 2014^23^	– 1392 participants from MESA cohort (asymptomatic individuals) – Separation based on sex: 640 males aged 64.71 ± 9.61 and 752 women aged 64.33 ± 9.81 – Follow-up 8.3 [7.5–8.6] years	– HARP method – LV Dyssynchrony based on time to peak systolic Ecc	– Women: 11 hard cardiac events (MI, aborted SCD, death from CAD), 17 all cardiac events and 17 strokes – Men:42 hard cardiac events (MI, aborted SCD, death from CAD), 62 all cardiac events and 42 strokes	– 1.5 T scanners – HARP 1.15; Diagnosoft
Venkatesh et al. 2014^24^	– 1544 patients from MESA cohort – Age 65 ± 9.7, 53% males – Follow-up 8.0 years	– HARP method – SRI – EDSR	– Heart failure (HF) and atrial fibrillation (AF) occurrence: 57 AF, 36 HF and 80 both	– 1.5 T scanners – HARP 1.15; Diagnosoft
Mordi et al. 2015^25^	– 539 consecutive patients, 56.8% referred to CMR due to HF – Age 48.1 ± 15.4, 63.6% males – Follow-up 2.2 ± 1.2 years	– HARP method – Ecc_global_ – LGE	– All-cause death, HF hospitalization and aborted SCD constituted the primary endpoint	– Argus software; Siemens, Erlangen, Germany – HARP version 5.03, Diagnosoft, Durham, North Carolina
Korosoglou et al. 2021^19^	– 1169 consecutive patients with HF, CAD, or clinically referred to stress CMR – Age 55 ± 17, 52% males – 61 healthy subjects aged 29 ± 9 and 51% males to render normal range of SENC strain – Follow-up 1.9 ± 0.4 years	– Fast SENC – Percentage of healthy myocardium based on Ecc global and GLS	– Hospitalization due to HF congestion and all-cause mortality constituted the primary endpoint – Initiation of HF treatment was the secondary endpoint of the study	– 1.5-T clinical scanners: Ingenia or Achieva, Philips Healthcare, Best, the Netherlands – Fast-Senc MyoStrain software (Myocardial Solutions, Inc., Morrisville, North Carolina, USA)
Steen et al. 2021^20^	– 111 patients referred to adenosine stress CMR – Age 62.6 ± 11.8, 68% males – Follow-up 1.94 ± 0.65 years	– Fast SENC – Global and regional Ecc, GLS and regionals longitudinal strain	– All-cause mortality, non-fatal MI and urgent revascularization with PCI or CABG constituted the primary endpoint of the study	– 1.5 T MR system (Achieva, Philips Healthcare, Best, The Netherlands – Fast-Senc MyoStrain software (Myocardial Solutions, Inc., Morrisville, North Carolina, USA)
Pezel et al. 2022^26^	– 1506 patients from MESA cohort – Age 63.3 ± 9.4, 54.6% males Follow-up of duration of 15.9 (12.9–16.6) years	– HARP method – Multilayer und total Ecc	– CVD including HF, congestive HF, MI, aborted SCD and CAD related death constituted the primary endpoint	– 1.5 T CVi, General Electric Medical Systems, Waukesha, WI; Sonata/Symphony Siemens Medical Solutions, Germany – MASS, 4.2 Medis, the Netherlands and HARP commercial v. 3.0, Myocardial Solutions/Diagnosoft, Morrisville, NC built in MATLAB (MathWorks, Natick, MA)

**Table 3:** Uni- and multivariate predictors of outcomes based on CMR derived myocardial strain.

Study	Myocardial strain and selected cut-off values	Univariate analysis	Multivariate analysis
Korosoglou et al. 2011^18^	– Visual SENC analysis (strain defect present or absent) – Strain reserve cut-off value = 0.94 – Strain rate reserve cut-off value = 1.75	Visual SENC, strain reserve and strain rate reserve enable diagnostic of anatomically relevant CAD and predict MACE (*p* < 0.001 for all)	– Visual SENC outperforms wall WMA for the prediction of outcomes – Visual and quantitative strain (strain rate reserve) provides incremental value to WMA for MACE prediction
Choi et al. 2013^22^	– Mid- and global Ecc – Mid Ecc < − 16.9%	Both Ecc_global_ and Ecc_mid_ predict HF (*p* < 0.001 for both)	– Adjusted for age, diabetes, hypertension LV mass index, ejection fraction, interim myocardial infarction and end systolic wall stress Ecc_global_ and Ecc_mid_ both predicted HF (*p*_*global*_ = 0.047 and *p*_*mid*_ = 0.015) – In second multivariable model without diabetes, LV ejection fraction and end systolic wall stress both Ecc_global_ and Ecc_mid_ remain statistically significant (*p*_*global*_ = 0.045 and *p*_*mid*_ = 0.007)
Sharma et al. 2014^23^	– LV Dyssynchrony based on SDTPEcc as a predictor for MACE Cut-off = 75th percentile of SD-TPS Separate analysis conducted by genders is provided	In women SDTPEcc predicted MI, HF, stroke, and death (*p* < 0.001), hard coronary events including MI, resuscitated cardiac arrest and CAD related death (*p* = 0.008), all-cause CAD (*p* = 0.002) and stroke or transient ischemic attack (*p* = 0.002). In men the results were not statistically significant	When adjusted for multiple parameters, SDTPEcc in women predicted MI, HF, stroke, and death (*p* = 0.015), hard coronary events including MI, resuscitated cardiac arrest and CAD related death (*p* = 0.026), stroke or transient ischemic attack (*p* = 0.013) but not all-cause CAD (*p* = 0.108). In men the results were not statistically significant
Venkatesh et al. 2014^24^	– The logarithm (Log) of SRI and EDSR Log(SRI) tertiles used as cut-off values	Log(SRI) was statistically significant for the prediction of HF and/or atrial fibrillation (AF) (*p* < 0.001). No further report is applicable due to lack of *p-*values in the results	No further report is applicable due to lack of *p-*values in the results
Mordi et al. 2015^25^	– Ecc_global_ (three groups based on two cut-offs values = − 11.21% and − 15.0%	Ecc_global_ predicted MACE (*p* < 0.001)	Ecc_global_ predicted MACE (*p* = 0.041) in whole population sample as well as in patients (N = 90) with cardiomyopathy or prior MI (*p* = 0.007)
Korosoglou et al. 2021^19^	– Normal myocardium based on percentage of segments with Ecc and longitudinal strain with a cut-off value of < − 17%	Prediction of hospitalization due to HF congestion and all-cause mortality in stage A and B HF patients with normal myocardium < 80% (*p* = 0.03) as well as of transition to incident HF (*p* < 0.001)	Not available
Steen et al. 2021^20^	Quantitative SAS (cut-off value = 6.5%)	Statistically significant for the primary endpoint with (*p* = 0.002)	Not available
Pezel et al. 2022^26^	Endo-, epi-, mid- and intramyocardial score of Ecc < 50% (based on − 10% and − 17% as cut-off values)	Strain from all segments (mid-, epi- and intra- *p* < 0.001 and endo *p* = 0.13) predicted HF incidence, as well as was congestive HF, MI, aborted sudden cardiac death and death due to CAD (*p* < 0.001 for all)	The epi-, mid- and intramyocardial score (*p*_endo_ = 0.56, *p*_mid_ = 0.004, *p*_epi_ < 0.001, *p*_intra_ < 0.001) predicted HF incidence, as well as of congestive HF, MI, aborted sudden cardiac death and death due to CAD by all layers (*p*_endo_ = 0.04, *p*_mid_ < 0.001, *p*_epi_ < 0.001, *p*_intra_ < 0.001) after adjustment of cardiovascular risk factors After further adjustment for heart-related medications the *p-*value remained statistically significant for all layers (*p* < 0.001)

*WMA* wall motion analysis, *Ecc* circumferential strain, *HF* heart failure, *LV* left ventricular, *SDTPEcc* time to peak systolic Ecc, *MI* myocardial infarction, *CAD* coronary artery disease, *SAS* segmental aggregate strain.

### Systematic review of studies including asymptomatic patients

Three studies analyzed data from the Multi-Ethnic Study of Atherosclerosis (MESA)^27^. Choi et al. investigated the CMR findings of 1768 individuals from the MESA cohort^22^. Circumferential strain (Ecc) predicted incident HF independent of clinical and CMR imaging parameters.

Similarly, time to peak circumferential systolic strain (SDTPEcc), a marker of LV-dyssynchrony, was predictive for MACE, including myocardial infarction, stroke, and death in asymptomatic female but not in male individuals (1392 included in total)^23^.

Another study, included 1544 patients from the MESA cohort, focusing on diastolic dysfunction^24^. Strain relaxation index (SRI) was predictive for the occurrence of heart failure and atrial fibrillation during follow-up.

The most recent study in asymptomatic patients was conducted by Pezel et al., including 1506 asymptomatic patients from the MESA study’s cohort^26,27^. Ecc was assessed using the HARP method^28,29^. Using Cox analyses, strain analysis was predictive for incident heart failure. The prognostic value remained after adjustment for traditional cardiovascular risk factors.

### Systematic review of studies including symptomatic patients

The first study in symptomatic patients was published in 2011^18^, included 320 patients, who underwent dobutamine stress CMR due to suspected or known CAD. 175 patients also underwent invasive coronary angiography. Visual and quantitative strain by SENC provided higher accuracy than cine-imaging for the detection of obstructive CAD and predicted MACE, independent of WMA and clinical parameters.

Using Fast-SENC, heart failure patients in stages A and B were prospectively analyzed^19^. Patients with reduced strain exhibited increased risk for death and hospitalization due to HF and for new onset of HF medications. In addition, strain analysis reclassified a substantial part of presumably healthy individuals at risk for heart failure to Stage B individuals with subclinical LV-dysfunction.

Another study by Steen et al., investigated 111 patients with known or suspected CAD who underwent adenosine stress CMR^20^. Patients with reduced strain showed higher rates for all-cause mortality, non-fatal MI, and coronary revascularization during follow-up.

The role of Ecc_global_ for the prediction of MACE in 539 symptomatic patients was investigated by Mordi et al.^25^ Reasons for referral of the patients were suspected dilative (36.9%) or ischemic cardiomyopathy (19.9%), myocarditis (11.9%), arrhythmias (13.9%), LV hypertrophy (10.9%), or aortic disease (6.5%). Hereby, Ecc_global_, LGE, and LVEF were predictive for MACE.

### Meta-analysis based on individual data in symptomatic patients

Individual baseline and CMR data were available in 1689 patients from 3 previous studies, (n = 200^20^, n = 320^18^, and n = 1169^19^, respectively) who underwent strain analysis by SENC/fast-SENC for ischemic heart disease (n = 985), suspected structural heart disease (n = 203), myocarditis or cardiotoxicity (n = 421), or due to other reasons (n = 80). Patients were 60.0 (47.4–70.8) years old and 976 (57.8%) were male, whereas 262 (15.5%) had diabetes mellitus (Table 4). Notably, the most recent study by Steen-H et al.^20^, reported on 111 patients with stress CMR and complete follow-up, while baseline data were available in 200 patients, which were included in the present analysis. Healthy volunteers^19^, were not included.

**Table 4:** Baseline and CMR characteristics.

Baseline and CMR data	All patients N = 1689
	**Baseline data**
Age (years)	60.0 (47.4–70.8)
Male gender	976 (57.8%)
Arterial hypertension	993 (58.8%)
Hyperlipidemia	783 (46.4%)
Diabetes mellitus	262 (15.5%)
History of CAD	548 (32.4%)
	**CMR data**
LV-ejection fraction (%)	55.6 (48.1–61.9)
Resting WMA	553 (32.7%)
CAD-related or non-ischemic LGE	701 (41.5%)
Resting WMA or LGE	780 (46.2%)
Pathologic strain values (abnormal baseline %normal myocardium < 80% or inducible strain defects)	1078 (63.8%)

*LV* left ventricular, *CAD* coronary artery disease, *WMA* wall motion abnormalities, *LGE* late gadolinium enhancement.

Follow-up was available in 1332 patients (n = 111^20^, n = 320^18^ and n = 901^19^, respectively) during a median follow-up duration of 22.4 (17.2–28.5) months. During follow-up, 19 (1.4%) patients died, 28 (2.1%) experienced non-fatal infarction, 52 (3.9%) underwent coronary revascularization and 86 (6.5%) were hospitalized due to heart failure.

**Figure 3:**
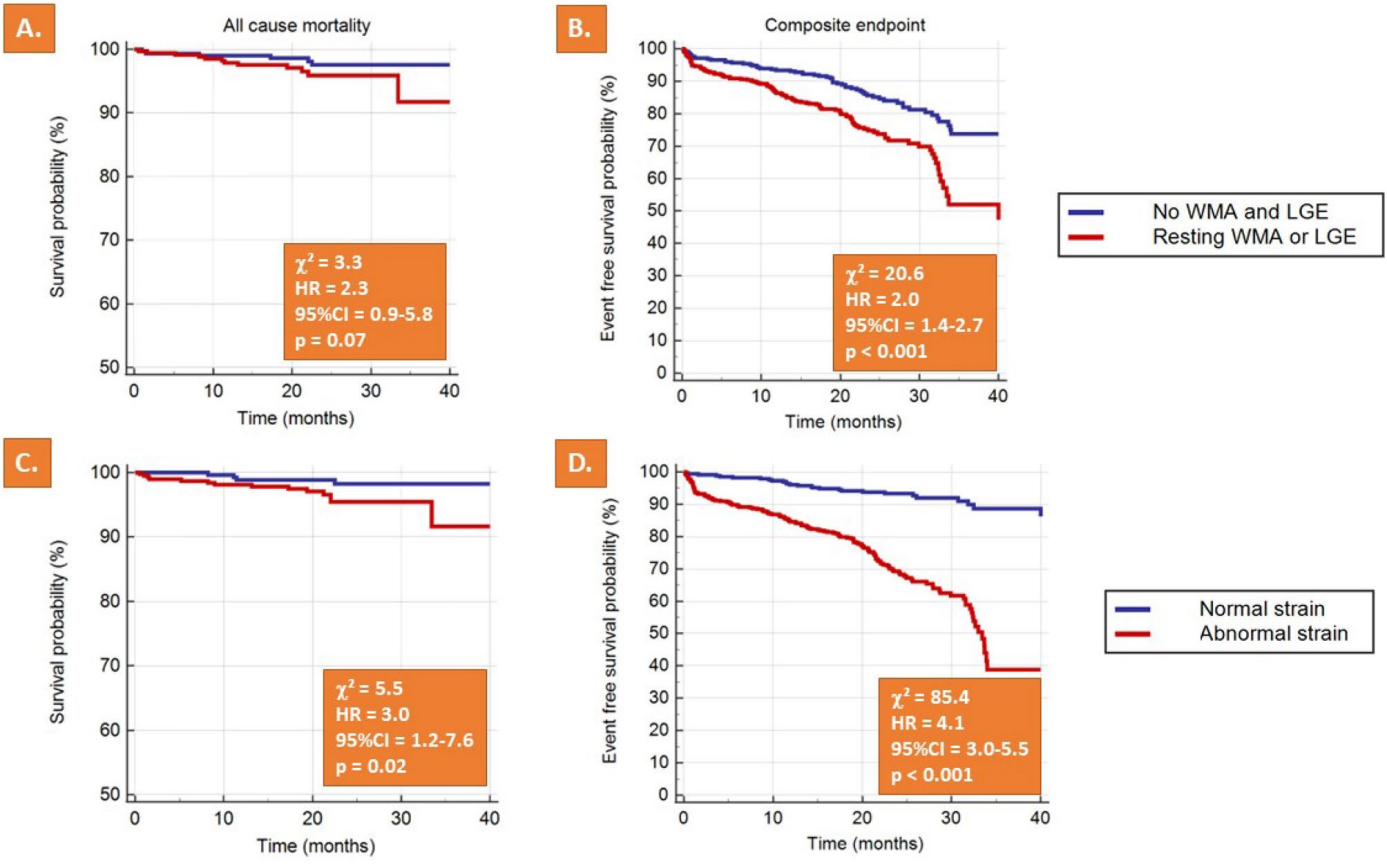
Resting WMA and LGE predicted the composite endpoint, whereas a non-significant trend was observed for all-cause mortality (**A**,**B**). Myocardial strain on the other hand, predicted both all-cause mortality and the composite endpoint (**C**,**D**).

Conventional CMR parameters such as resting WMA and LGE predicted the composite endpoint, whereas a non-significant trend was observed for all-cause mortality (HR of 2.0, *p* < 0.001 and of 2.3, *p* = 0.07, Fig. 3A,B). Myocardial strain by SENC/fast-SENC, on the other hand, predicted both all-cause mortality and the composite endpoint with high accuracy (HR of 3.0, *p* = 0.02 and of 4.1, *p* < 0.001, Fig. 3C,D).

**Figure 4:**
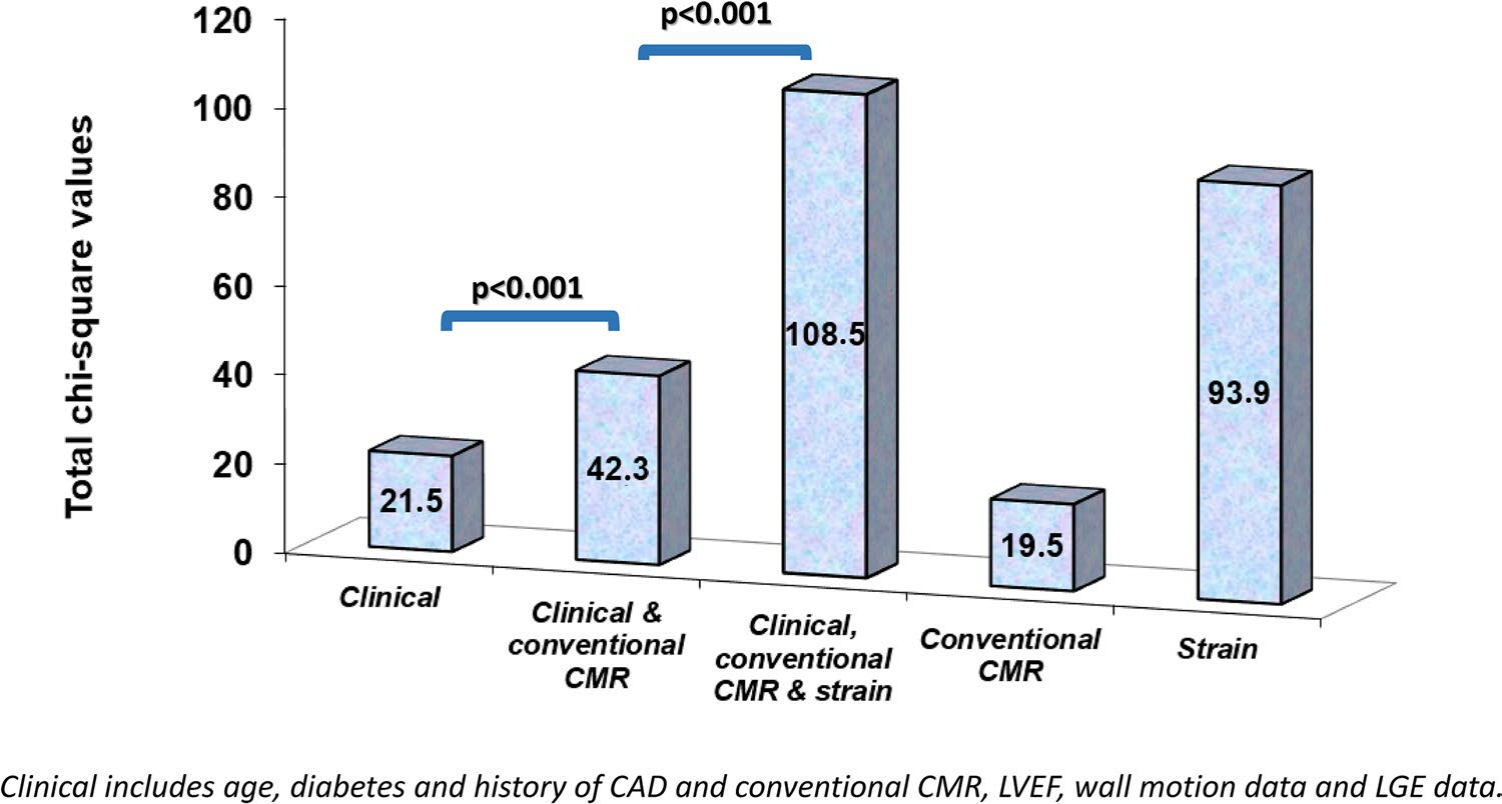
Hierarchical Cox-proportional hazard regression models pointed to the incremental value of CMR paraments beyond clinical variables and of myocardial strain beyond conventional CMR paraments (*p* < 0.001 for both).

Using Cox-proportional hazard regression models, myocardial strain by SENC/fast-SENC provided the most robust prediction of the composite endpoint, independent of clinical and conventional CMR variables (Table 5). Hierarchical Cox-proportional hazard regression models demonstrated the incremental value (i) of CMR paraments compared to clinical data and (ii) of myocardial strain compared to conventional CMR markers (Fig. 4).

**Table 5:** Cox-proportional hazard regression models for prediction of the composite endpoint.

Covariates	b	SE	Wald	*p*-values	Exp(b)	95% Cl of Exp(b)
Age	0.013	0.0058	5.17	0.02	1.01	1.00–1.02
Diabetes mellitus	0.29	0.16	3.02	0.08	1.34	0.96–1.86
LV-ejection fraction (%)	− 0.004	0.007	0.26	0.60	0.99	0.98–1.01
Resting WMA or LGE by CMR	0.43	0.17	6.25	0.01	1.55	1.09–2.18
Pathologic strain values (abnormal baseline %normal myocardium < 80% or inducible strain defects)	1.49	0.20	50.91	< 0.0001	4.46	2.96–6.73

## Discussion

Our study summarizes for the first time in the current literature the value of CMR based strain by HARP, SENC and fast-SENC in the clinical setting, serving as a promising surrogate parameter for the prediction of MACE in patients both with ischemic and non-ischemic cardiac diseases as well as in asymptomatic individuals^18–20,22–26^. By performing meta-analysis of individual patient data, SENC/fast-SENC provided the most robust prediction of the composite endpoint of death, myocardial infarction, coronary revascularization and hospitalization due to heart failure, beyond clinical parameters and conventional CMR parameters, such as WMA, LV-ejection fraction and LGE (HR of 4.46, 95% CI = 2.96–6.73, *p* < 0.001)^18–20^. In addition, the assessment of myocardial strain by SENC/fast-SENC predicted all-cause mortality (HR of 3.0, 95% CI = 1.2–7.6, *p* = 0.02).

The MESA study offers a large database with healthy patients undergoing multiple examinations including CMR, whereas long-term follow-up of up to 8 years was available in the studies included in our systematic review^22–24,26^. Various strain variables assessed by HARP emerged as significant predictors of MACE and incident heart failure in asymptomatic individuals from different ethnicities, surpassing the value of clinical parameters and standard CMR variables^22–24,26^. The underlying pathophysiologic mechanism of this observation is not completely understood. Possibly, regional circumferential myocardial dysfunction represents a response to increased wall stress, reflecting local alterations of myocardial properties, such as fibrosis or ischemia due to microvascular disease or CAD. This increased afterload may contribute to the development of progressive myocardial remodeling and dysfunction, triggering poorer outcomes^30^. In addition, Ecc was significantly related to the LV mass index, which again underlines that the relationship between reduced strain and subclinical heart failure, which may convert to symptomatic disease due to adverse remodeling of the ventricle^30,31^. Notably, the thresholds provided for strain values in studies including asymptomatic patients have been largely inhomogeneous, ranging between − 10% and − 17%, whereas some studies selected relative cut-off values based on percentiles or tertiles. In addition, follow-up duration largely ranged between 2.2 and 15.9 years in these studies.

Four studies on the other hand, focused on the ability of SENC/fast-SENC for the prediction of outcomes in patients who underwent clinically indicated CMR examinations^18–20,25^. In two of these studies focusing on symptomatic patients with CAD, SENC and fast-SENC respectively, outperformed the ability of WMA for the diagnostic classification and risk stratification of patients with ischemic heart disease^18,20^. The results were similar although different stressors (dobutamine versus adenosine) were used for pharmacologic stimulation, which underlines the wide applicability of SENC for ischemia detection. In the two further studies, investigating patients who underwent clinically indicated CMR due to suspected ischemic and non-ischemic, structural cardiac diseases, the role HARP and fast-SENC for risk stratification was reestablished^19,25^. Hereby, patients with normal myocardium > 80% by fast-SENC exhibited better outcomes compared to patients with reduced baseline strain, who experienced higher mortality, higher rates for hospitalization due to heart failure symptoms and significantly more frequent transition rates from subclinical LV-dysfunction to symptomatic heart failure^19^. In addition, in our meta-analysis, based on individual patient data, SENC and fast-SENC provided the most robust prediction of MACE beyond clinical and conventional CMR parameters, exhibiting incremental value for the risk stratification of patients with a broad spectrum of cardiac diseases^18–20^. In addition, myocardial strain achieved prediction of all-cause mortality, which was not the case with conventional CMR markers.

### Comparison to myocardial strain assessment by feature tracking imaging (FTI) and technical considerations

Several previous studies investigated the role of feature tracking imaging (FTI) for the risk stratification of patients with ischemic and non-ischemic heart disease^32–36^. In this regards, FTI derived GLS exhibited incremental value to CMR variables such as LV-ejection fraction and late gadolinium enhancement (LGE) for the prediction of MACE, including sudden cardiac death, resuscitated cardiac arrest and hospitalization due to heart failure in patients with hypertrophic cardiomyopathy^32^. In addition, LV strain parameters were independent predictors of MACE beyond clinical and conventional CMR markers, such as LVEF and LGE, in 162 patients with acute myocarditis, analyzed within a multi-center trial, while left atrial and right ventricular strain were less useful in this context^33^. In the same direction, previous studies underlined the incremental prognostic value of FTI in patients with non-ischemic dilative cardiomyopathy, beyond NYHA classification, LV-ejection fraction and LGE^34^. This could be confirmed in recent multi-center CMR studies, where FTI derived strain parameters surpassed the value of conventional functional CMR parameters, thus strengthening the body of evidence for the clinical implementation of strain for the risk stratification of patients with non-ischemic heart diseases^35,36^. Fewer studies, however, have investigated the value of FTI for the diagnostic classification or risk stratification of patients with ischemic heart disease^37,38^.

From a technical point of view FTI is based on pattern matching techniques across multiple images in a cardiac cycle^39^. By FTI, pixels are identified in one frame and followed in the next frames, enabling tracking of myocardial deformation with conventional cine images^40^. This is the foremost advantage of FTI since it does not require additional image acquisition and can estimate myocardial strain using clinical SSFP cine images. Different software packages with FTI however, use different algorithms for the calculation of strain, which results in different numerical values. These values are also different from CMR based HARP or SENC and fast-SENC and considerations have been raised, regarding strain over- or underestimation with FTI, which may be less sensitive in terms of disease detection^41,42^. In addition, strain reproducibility may be lower by FTI, compared to SENC, which may allow more comprehensive assessment of regional myocardial strain compared to FTI^43,44^. Such differences may be decisive for the diagnostic classification or risk stratification of patients with ischemic heart disease^38,45^. In this regard, FTI based strain exhibited lower precision than fast-SENC for the identification of segments with regional myocardial dysfunction due to ischemic heart disease^46^.

Considering the practical advantages of fast-SENC compared to HARP and SENC, it should be noted, that fast-SENC can be acquired during free-breathing of the patients, within a single heartbeat and high heart rates under inotropic stress CMR (> 150 bpm), which is of clinical importance, especially in patients with symptomatic heart failure, arrhythmias and chronic obstructive lung disease^47^. In addition, post-processing analysis with fast-SENC requires much lower time spent, compared to earlier sequences like HARP, thus increasing the potential of fast-SENC for translation into the clinical realm^44,47^. Finally, the use of artificial intelligence (AI) in CMR imaging protocols evaluating potential clinical predictors in patients with cardiovascular diseases continuously increases^48^. Incorporating AI in future studies may also increase the precision of strain algorithms for the risk stratification of patients, simultaneously reducing the required time spent for quantification analysis.

### Limitations

Our study has some limitations. Thus, a classical meta-analysis was not possible due to substantial heterogeneity in the definition of outcomes between trials. We therefore performed individual patient data analysis in only 3 of the studies. In this regard, the strain sequences and acquisitions differed between the 3 studies, preventing the selection of a binary illustration and a universal cut-off value. In addition, studies were conducted with different scanners and different image quality can be anticipated, which may have affected the resultant image quality and the acquired strain values. However, our study cohort included patients with cardiac diseases based on largely heterogeneous etiologies, so that it may add important evidence for the value of myocardial strain for the risk stratification of symptomatic patients across a wide range of cardiac disorders. In addition, WMA and LGE were assessed visually in studies building the base for our meta-analysis, whereas data on T1 and T2 mapping and extracellular volume fractions (ECV) were not available. However, myocardial strain was also treated as a categorical variable in our statistical analysis, although quantification or semi quantification analysis has been available in the individual studies. In addition, T1 and T2 mapping techniques, although meanwhile established for the diagnostic work of patients with non-ischemic cardiomyopathies^8,9,49^, were not widely used in studies performed more than one decade ago.

## Conclusions

Reduced myocardial strain derived from myocardial tagging by HARP, SENC or fast-SENC is a predictor of all-cause mortality and adverse outcomes in symptomatic patients with a wide range of ischemic or non-ischemic cardiac diseases as well as in asymptomatic individuals. Its value in terms of diagnostic classification and risk stratification is shown across multiple scanner and software vendors. SENC and fast-SENC provide the most robust prediction of MACE, beyond clinical parameters and conventional CMR parameters, such as WMA, LV-ejection fraction, and LGE. Especially in patients where contrast agent administration may be problematic, fast-SENC, which allows single heartbeat acquisitions of myocardial strain, and may represent a valuable alternative for the quantitative characterization of underlying myocardial pathologies.

### Supplementary Information

Supplementary Information.

## Data Availability

The datasets used and/or analysed during the current study available from the corresponding author on reasonable request.
